# Make Short Work of It

**Published:** 1897-04

**Authors:** 


### MAKE SHORT WORK OF IT.

A bill has been presented in the State Legislature of Pennsylvania by Mr. Jennings, of Sullivan County, proposing to reopen the law of 1889, requiring registration of all veterinarians. The object of the measure is to enable those who have been in practice or who have been using the title for the past ten years to be privileged to register on or before January 1, 1898. As this Act is disguised legislation of a special nature and of the most pernicious character, every veterinarian in the State should raise his voice against it, wield his pen to defeat it, and, in making short work of it, impress his representative that there will be no toleration of such vicious legislation as this which is so prejudicial to public good.

---

ANOTHER German embargo is announced involving an eight-day quarantine of all horses from the United States. But the order does not apply to those from Canada. After this period, on passing veterinary inspection, they are permitted to enter the markets for sale. Forbearance with the annoyances aimed at this country has ceased to be a virtue, and only retaliatory measures will wake these people up. Stop the beet-root importation, analyze some of the beers and wines sent us, and there will soon be a stay of proceedings.

---

NEW YORK CITY has listed tuberculosis among the infectious and contagious diseases, and proposes to deal with it from such a standpoint, for all of which every true student of sanitary medicine should be deeply thankful, as it cannot help to solve much of the mystery surrounding the most potent methods of propagation and transmission, and it will be a means of determining how radical must be the measures to be adopted as sanitary police regulations.

---

THE State Grange of Pennsylvania strongly indorses the proposed legislation to prevent the introduction of untested cows from other States among our dairy herds. It will also petition the Legislature to appropriate a sum of money for prosecuting the work of investigation of several of the diseases so destructive to the live-stock interests of the State, and ask that suitable facilities may be provided and that the work be under the direction of the State Live-stock Sanitary Board. All of which is in keeping with the proper appreciation of the live-stock and dairy interests of the Keystone State.

